# Asymptomatic Leukocytospermia and Assisted Reproductive Technology Outcomes: Reason for concern?

**DOI:** 10.1590/S1677-5538.IBJU.2025.0166

**Published:** 2025-04-15

**Authors:** Marie-Christin Reich, Natalie Heide, Peter Humaidan, Sandro C. Esteves

**Affiliations:** 1 Skive Regional Hospital Fertility Unit Skive Denmark Skive Regional Hospital, Fertility Unit, Skive, Denmark; 2 Aarhus University Faculty of Health Department of Clinical Medicine Aarhus Denmark Department of Clinical Medicine, Faculty of Health, Aarhus University, Aarhus, Denmark; 3 ANDROFERT Clínica de Andrologia e Reprodução Humana Campinas SP Brasil ANDROFERT, Clínica de Andrologia e Reprodução Humana, Campinas, SP, Brasil; 4 Universidade Estadual de Campinas Departamento de Cirurgia, Disciplina de Urologia Campinas SP Brasil Departamento de Cirurgia, Disciplina de Urologia, Universidade Estadual de Campinas - UNICAMP, Campinas, SP, Brasil

**Keywords:** Infertility, Reproductive Techniques, Assisted, Oxidative Stress

## Abstract

Leukocytospermia, defined as ≥1×10^6^ white blood cells (WBC)/ml of semen, is a condition frequently observed in infertile men. While symptomatic leukocytospermia is often associated with genital tract infections and managed accordingly, the clinical significance of asymptomatic leukocytospermia remains uncertain—particularly in the setting of Assisted Reproductive Technology (ART). Seminal leukocytes, primarily neutrophils, play a physiological role in immune surveillance and tissue homeostasis. However, when excessively activated, they may generate high levels of reactive oxygen species (ROS), contributing to oxidative stress, sperm dysfunction, and DNA damage. This narrative review critically examines whether asymptomatic leukocytospermia adversely affects ART outcomes, including fertilization, embryo development, clinical pregnancy, and live birth rates. A synthesis of current evidence—including meta-analyses and large retrospective studies—suggests that asymptomatic leukocytospermia does not negatively impact these outcomes. Moreover, standard sperm preparation techniques and the widespread use of ICSI appear to neutralize any potential deleterious effects from seminal leukocytes. Given the absence of compelling evidence supporting its harmful impact on ART success, routine treatment of asymptomatic leukocytospermia—particularly with empiric antibiotics—is not recommended. Such interventions may disturb the natural immune balance, promote antibiotic resistance, and increase healthcare burdens without demonstrable benefit. Nonetheless, selective treatment may be justified in specific scenarios, such as recurrent implantation failure or early pregnancy loss. Further research is warranted to standardize leukocyte detection methods and to clarify the role of adjunctive therapies. Until more definitive data emerge, an individualized, evidence-based approach remains the most appropriate strategy for managing asymptomatic leukocytospermia in infertile men pursuing ART.

## INTRODUCTION

Infertility affects about 17% of couples worldwide (1), with male factors contributing to 20-30% of the cases (2, 3). Among the potential causes of male infertility, leukocytospermia –defined as an increased concentration of white blood cells (WBCs) in semen– remains a subject of debate. The reported prevalence of leukocytospermia varies widely, ranging from 2 to 40% among infertile men, depending on the study population, detection method, and diagnostic threshold used (4–6).

The World Health Organization (WHO) defines leukocytospermia as the presence of ≥1 × 10^6^ WBC/mL of semen (4, 7). It can result from an infection, such as male genital tract infection (MGTI), including male accessory gland infection (MAGI), which is typically managed with antibiotics and frequent ejaculation (8, 9). However, non-infectious causes –including non-bacterial inflammation, autoimmune disease, varicocele, and unhealthy lifestyle factors such as tobacco use or chronic alcohol consumption– are also implicated (5, 8, 10–13). In such cases, treatment strategies may involve anti-inflammatory medications, antihistamines, antioxidants, and lifestyle modifications (5, 14–16).

The term seminal leukocytes collectively refers to WBCs found in semen, which consist of 2-5% T-lymphocytes, 20-30% macrophages, and 50-60% granulocytes, primarily neutrophils (17, 18). These cells originate from the testis, epididymis, and prostate (19–21). Under normal physiological conditions, seminal leukocytes play a key role in immune surveillance, helping to regulate inflammatory responses by releasing cytokines and proinflammatory mediators. This immune activity facilitates pathogen elimination and supports reproductive health by removing abnormal and immature sperm cells through phagocytosis and the release of reactive oxygen species (ROS) (22, 23).

In cases of infection or inflammation, leukocytes are actively recruited through chemotaxis, which directs them from the bloodstream to affected tissues (24). Once at the site of inflammation, leukocytes become activated by integrins and cytokines, including tumor necrosis factor alpha (TNFα) and interleukins (25, 26). This activation leads to the release of large amounts of proinflammatory cytokines and ROS, resulting in oxidative stress, which can damage sperm plasma membranes and DNA, ultimately compromising sperm quality and contributing to male infertility (27–30).

A key distinction must be made between symptomatic and asymptomatic leukocytospermia. Symptomatic leukocytospermia is associated with MGTI or MAGI and is often accompanied by clinical symptoms like urogenital pain, dysuria, pollakiuria, or other urine tract disturbances (31–33). Diagnosis typically involves identifying the causative pathogens to guide targeted antibiotic therapy (31–33). Conversely, asymptomatic leukocytospermia presents without overt clinical symptoms and may have infectious and non-infectious origins (6, 34). Consequently, the necessity for treatment in cases of asymptomatic leukocytospermia remains a topic of ongoing debate (6, 35), particularly when evaluating its role in the context of Assisted Reproductive Technology (ART) outcomes (36).

European and American Urological guidelines provide no clear recommendations on managing asymptomatic leukocytospermia in men with infertility (37, 38). In modern healthcare systems, the cost-effectiveness of ART, including in vitro fertilization (IVF) and intracytoplasmic sperm injection (ICSI), depends on treatment expenses, success rates, and risk of multiple pregnancies (39, 40). Given that leukocytospermia has been associated with impaired semen quality, it is crucial to determine whether treatment improves ART outcomes and enhances the cost-effectiveness of fertility care for affected couples.

This article critically assesses whether an evidence-based rationale exists for treating asymptomatic leukocytospermia in the context of ART, specifically in IVF and ICSI cycles. By systematically examining the existing literature, we seek to clarify whether intervention is necessary to optimize ART success or whether asymptomatic leukocytospermia poses minimal concern.

## LEUKOCYTOSPERMIA AND MALE INFERTILITY

### Inflammation, Leukocytospermia, and the Impact on Sperm Function

Infection-induced inflammation triggers an immune response that activates local macrophages and recruits leukocytes from the bloodstream to the site of infection (41). The testicular immune defense protects male germ cells while allowing an inflammatory response to combat infections (42).

Macrophages play a key role in immune regulation through phagocytosis and the secretion of proinflammatory and anti-inflammatory cytokines (25, 43). These antigen-presenting cells are critical for immune homeostasis, spermatogenesis, and regulation of autoimmunity against testicular antigens (44, 45).

Another part of the natural immune defense system is leukocytes, especially neutrophil granulocytes. In a healthy state, circulating neutrophils are resting and most are eliminated without receiving an activating signal (46). However, when infections or injuries occur, they must be able to respond appropriately as multifunctional first responders (46). Neutrophils that encounter a series of agonists enter a pre-activated or primed state that sets them on high alert, enabling them to respond aggressively (e.g., through degranulation, respiratory burst activity, increased phagocytosis, release of ROS, and bioactive mediators) if another activation stimulus occurs (46). Due to the variety of host- and pathogen-derived mediators, priming can be induced by multiple signaling pathways and intracellular processes, such as chemokines, cytokines, alarmins, integrins, pathogen‐derived molecules, and mechanical forces (46). The signaling pathways and the resulting cellular phenotype depend on the priming agent acting on the neutrophils and the specific environment (46).

Despite their critical role in the defense system, leukocytes can also become detrimental when activated as they produce 1,000 times more ROS than spermatozoa (47). While controlled ROS levels are essential for sperm maturation, capacitation, acrosome reaction, and chromatin condensation (48), excessive ROS generation by activated seminal leukocytes depletes seminal antioxidants (49) such as catalase, glutathione, and superoxide dismutase, which scavenge free radicals (49–51). This imbalance leads to oxidative stress, potentially damaging spermatozoa (52).

ROS, including hydroxyl radicals (-OH), superoxide anions (O2-), and hydrogen peroxide (H2O2), are potent oxidants (53–57). Importantly, spermatozoa are particularly vulnerable to oxidative stress due to the high content of polyunsaturated fatty acids in their plasma membrane (53). While H2O2 can penetrate plasma membranes and cause intracellular damage, O2- and -OH primarily induce lipid peroxidation, disrupting membrane integrity and impairing sperm function (28, 58–60). ROS-mediated intracellular damage ranges from chromatin cross-linking and protein impairment to DNA modifications and fragmentation (56, 61–65).

**Figure 1:**
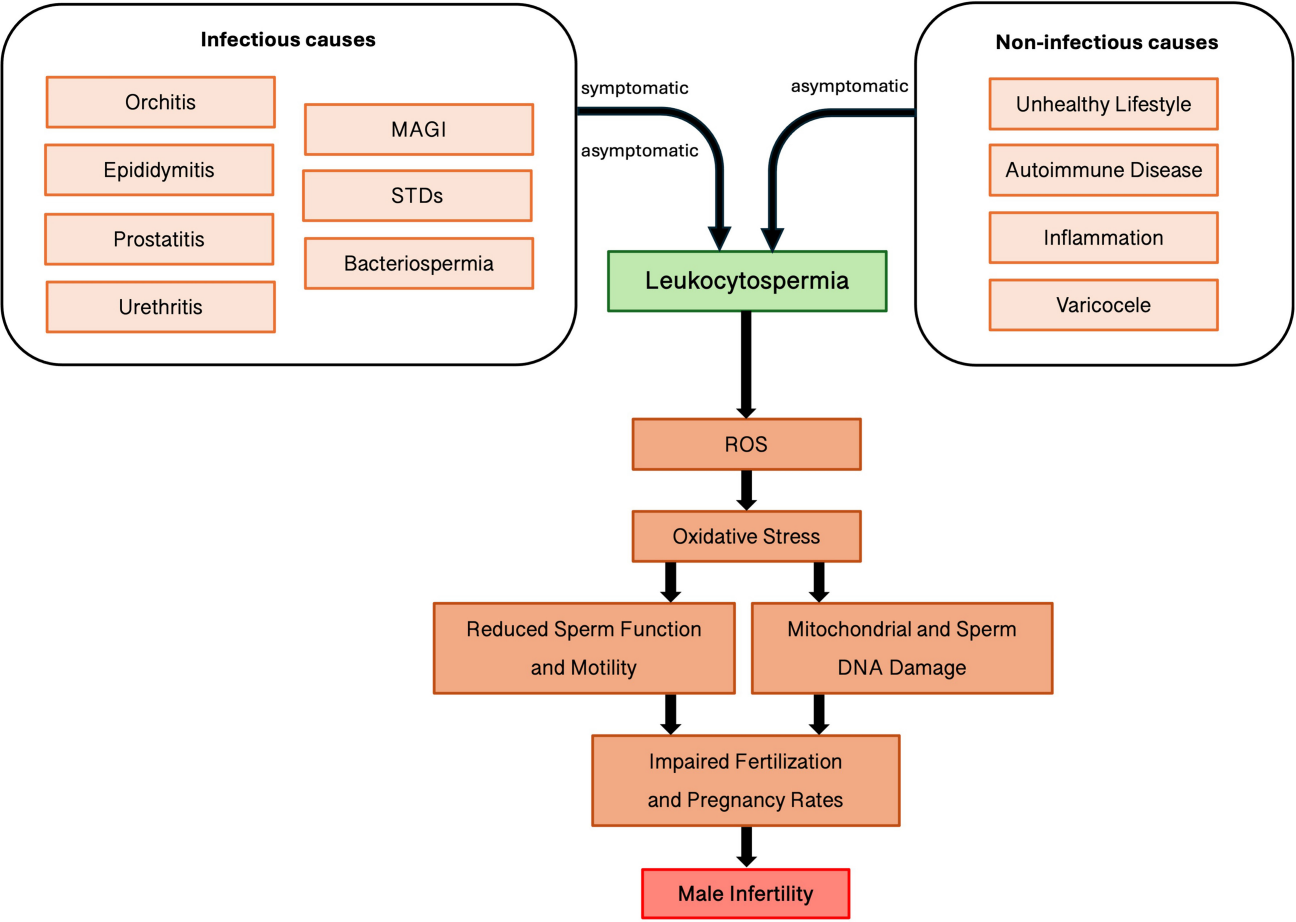
Pathophysiological Pathways Linking Infectious and Non-Infectious Causes of Leukocytospermia to Male Infertility via Oxidative Stress. MAGI, male genital infections; STDs, sexually transmitted diseases; ROS, reactive oxygen species.

Oxidative stress further compromises sperm function by reducing acrosine activity, impairing sperm-oocyte fusion (66–69), and damaging mitochondrial function (62, 70, 71). Since mitochondria are crucial for adenosine triphosphate (ATP) production in the sperm cell, oxidative damage to mitochondrial DNA (mtDNA) can impair sperm motility and overall fertilization potential (62, 70–76). Additionally, ROS-induced axonemal damage directly affects sperm motility (43, 77) (Figure-1).

Maintaining sperm DNA integrity is essential for successful fertilization, embryo development, and ongoing pregnancy (78). Increased sperm DNA fragmentation is associated with prolonged time to pregnancy in fertile couples (79). While sperm with fragmented DNA can fertilize oocytes, early embryonic development often arrests when paternal genes are not correctly functional (80). Additionally, sperm DNA damage may increase the risk of chromosomal abnormalities and miscarriages (81), reducing success rates of both natural conception and ART (82–88).

Given that leukocytospermia might increase the proportion of spermatozoa with impaired DNA compared to non-leukocytospermic samples (78), a question to ask is: should screening for leukocytospermia become a routine part of infertility assessments for couples undergoing ART? This question is relevant to refine clinical guidelines for male infertility evaluation and ART decision-making.

### Detection Methods of Seminal Leukocytes

Several techniques are available for detecting seminal leukocytes, each with varying levels of specificity and practicality. One traditional method involves staining a sperm smear using the Papanicolaou technique, which distinguishes leukocytes from spermatids and spermatocytes based on differences in staining properties, size, and nuclear morphology (89). However, the method is prone to morphological misidentification, making it less reliable (89).

A more commonly used approach is the histochemical peroxidase test, which quantifies leukocytes containing the peroxidase enzyme –a characteristic feature of granulocytes (89). This test is quick, inexpensive, and widely used for initial screening. However, it has limitations: it cannot detect activated polymorphonuclear cells that have already released their granules or peroxidase-negative leukocytes such as lymphocytes, macrophages, and monocytes (89). Therefore, the number of total leukocytes in semen may be underestimated, though peroxidase-positive granulocytes remain the predominant leukocyte type in semen (89).

A more precise alternative is the immunocytochemical method, which uses monoclonal antibodies targeting the CD45 antigen, a pan-human leukocyte marker (89). This approach enables the detection of all leukocyte subtypes, including granule-released polymorphonuclear leukocytes and peroxidase-negative cells such as lymphocytes, macrophages, and monocytes. However, while more accurate, immunocytochemical staining is also more time-consuming and expensive compared to histochemical methods (89).

## LEUKOCYTOSPERMIA AND ART OUTCOMES: INSIGHTS FROM META-ANALYSES AND RECENT STUDIES

### Meta-analysis

A 2020 systematic review and meta-analysis examining the impact of leukocytospermia on sperm quality and ART outcomes analyzed 28 case-controlled retrospective studies. The findings revealed no significant differences in fertilization rates (FR) or clinical pregnancy rates (CPR) between couples with and without leukocytospermia undergoing conventional IVF or ICSI (90). However, males with leukocytospermia exhibited significantly lower sperm concentration and reduced progressive motility, indicating a negative impact on sperm parameters. Notably, two-thirds of the included studies (n=18) did not differentiate between symptomatic and asymptomatic leukocytospermia (90). The ten remaining studies specifically compared asymptomatic leukocytospermia to non-leukocytospermic controls. The meta-analysis indicated a considerable inter-study heterogeneity due to differences in the distinction between symptomatic and asymptomatic cases and in the detection methods across studies, which introduced potential inconsistencies in reliability and accuracy, limiting the generalizability of the findings (90).

To address this heterogeneity, the meta-analysis included a subgroup analysis based on leukocyte detection methods, categorizing studies into those using CD45-based immunocytochemistry or flow cytometry, peroxidase staining, and morphological evaluation of stained semen smears (90). Another subgroup analysis included only studies assessing asymptomatic leukocytospermia without genital tract infections (90). After adjusting for these variables, the previously observed association between leukocytospermia and reduced sperm concentration and progressive motility was no longer significant, suggesting that methodological differences and confounding factors contributed to the initial findings (90). Moreover, most studies did not account for key variables such as duration of sexual abstinence and patient age, both of which are known to influence semen quality (90).

Ultimately, after controlling for confounding factors, the meta-analysis concluded that asymptomatic leukocytospermia did not negatively impact basic semen parameters or sperm DNA integrity (90). Furthermore, subfertile couples with asymptomatic leukocytospermia did not exhibit reduced reproductive outcomes after ART, reinforcing the notion that leukocytospermia alone may not be a critical determinant of ART success (90).

### Large-Scale Retrospective Studies

The largest retrospective study included in the meta-analysis discussed above had some divergent findings (91). The authors compared conventional IVF and ICSI outcomes among three groups: non-leukocytospermia (n=3,026), low-level leukocytospermia (<10^6^ WBC/mL, n=344), and high-level leukocytospermia (≥10^6^ WBC/mL, n=138) (91). No significant differences were found in pregnancy complications or congenital malformations across the groups (91). However, leukocytospermic patients underwent significantly more ICSI cycles than non-leukocytospermic patients (91). Interestingly, couples with leukocytospermia had more cycles with at least one high-quality embryo and more two pronuclei (2PN) zygotes than those without leukocytospermia (91).

Despite these favorable laboratory parameters, total sperm concentration and total sperm count were significantly lower in leukocytospermic men, although sperm vitality was slightly higher in the low-level leukocytospermic group (91). Notably, FR and CPR were significantly higher in the presence of leukocytospermia (91). Based on these findings, the authors suggested that leukocytospermia may be physiologically advantageous to a certain extent (91). They further hypothesized that ROS play a dual role, being essential for various physiological processes such as DNA condensation, sperm capacitation, and acrosome reaction, but potentially detrimental when present at excessively high concentrations over prolonged periods (91).

However, a notable finding was that high-level leukocytospermia was associated with increased early pregnancy loss and a three-fold higher risk of ectopic pregnancy compared to the non-leukocytospermia group (91). These differences remained significant even after excluding women with reproductive tract anomalies and ovarian dysfunction, though only the increase in early miscarriages reached statistical significance (91). The authors suggested that while ROS can impair various sperm functions, they do so to various degrees. Although high ROS levels contribute to DNA fragmentation, both sperm fusion and motility remain primarily unaffected, potentially explaining the increased fertilization rates alongside higher early pregnancy loss rates in the high leukocytospermia group (91).

Overall, the study suggested that low-level leukocytospermia may enhance sperm fertilization capacity and pregnancy outcomes, while high-level leukocytospermia, despite not impairing sperm fertilizing ability, may compromise early pregnancy (91). Yet, the study had several limitations. It made no distinction between symptomatic and asymptomatic leukocytospermia, relied on the peroxidase test for WBC detection, and had a retrospective design (91). Despite these limitations, male and female ages and indications for IVF/ICSI were comparable across groups, supporting the validity of the findings (91).

### Additional Retrospective Studies on ART Outcomes

Another retrospective study comparing IVF, ICSI, and split insemination outcomes between leukocytospermic and non-leukocytospermic men reported no significant differences in FR, CPR, or live birth rates (LBR) (92). However, in conventional IVF cycles, sperm concentration and progressive motility were significantly lower in leukocytospermic men (92). Similarly, leukocytospermic patients undergoing ICSI exhibited reduced progressive motility (92).

The study further analyzed split insemination cycles (i.e., using IVF and ICSI simultaneously) to determine whether the insemination method influenced ART outcomes in the presence of leukocytospermia. No differences were observed in FR, CPR, and LBR between IVF and ICSI within the leukocytospermia group. However, ICSI resulted in more 2PN zygotes, available embryos, and good-quality embryos compared to IVF (92).

While informative, the study had limitations, including its retrospective design and relatively small sample size (n=133; leukocytospermic men: 63 IVF, 38 ICSI, 32 split insemination cycles) (92). Moreover, the peroxidase test was used for WBC detection, and there was no differentiation between symptomatic and asymptomatic leukocytospermia (92). Semen samples were collected after a wide range of ejaculatory abstinence intervals (2-7 days). Nevertheless, all included patients underwent their first ART cycle and had a normal karyotype. Women older than 40 years and those with uterine malformations, coagulation, or thrombophilia disorders were excluded (92). The study supported that leukocytospermia does not compromise ART success, though ICSI may provide certain embryological advantages over conventional IVF (92).

### Leukocytospermia and Preimplantation Genetic Testing Outcomes

A 2024 retrospective study examined the impact of leukocytospermia on ICSI outcomes with preimplantation genetic testing for aneuploidy (PGT-A). Among 5,435 fertilization cycles, no significant differences were observed in LBR, FR, 2PN rate, or embryo aneuploidy rates between couples with and without leukocytospermia (93). Moreover, leukocyte concentrations did not predict LBR, thus also suggesting that leukocytospermia does not negatively impact ART outcomes (93).

The authors proposed that the unique setting of ICSI with PGT-A might have mitigated any potential adverse effects of leukocytospermia, as sperm selection during ICSI could reduce the influence of leukocyte-mediated oxidative stress (93). However, they cautioned that these findings might not directly translate to conventional IVF treatments (93).

As with prior studies, leukocytospermia was detected using the peroxidase test, and no distinction was made between symptomatic and asymptomatic cases (93). However, semen samples were collected after a well-defined 2-5 days of ejaculatory abstinence, and female age did not significantly differ between groups, ensuring comparability (93).

### The Case Against Routine Treatment of Asymptomatic Leukocytospermia

Given the lack of conclusive evidence linking asymptomatic leukocytospermia to impaired ART outcomes, routine treatment remains controversial. Below are the primary arguments against intervention.

1. There is no significant influence on ART success
  - Studies show no significant differences in ART success rates between men with and without asymptomatic leukocytospermia (90).
  - Sperm preparation methods (e.g., density gradient centrifugation) can eliminate leukocytes, reducing their potential impact on fertilization and embryo development (94, 95).
  - ICSI appears to circumvent potential sperm quality issues, reducing any need for routine treatment (89, 92, 93).
  - Asymptomatic leukocytospermia may resolve naturally over time without medical intervention (35).
  - There is no evidence that treating asymptomatic leukocytospermia improves conception rates (38, 96).
2. The Immune Surveillance Hypothesis
  - Seminal leukocytes may serve a beneficial function, defending against subclinical infections and oxidative stress (17, 20).
  - Treating asymptomatic cases could disturb the natural immune balance without offering clear reproductive benefits (97).
3. Risks of Overtreatment
  - Unnecessary antibiotic use contributes to antimicrobial resistance (97).
  - Antibiotics may alter the seminal microbiome, potentially affecting fertility (35, 94, 98, 99).
  - Overdiagnosis and overtreatment can lead to increased costs and unnecessary stress for couples undergoing ART (100).

### Clinical Justification for Targeted Treatment

**Figure 2:**
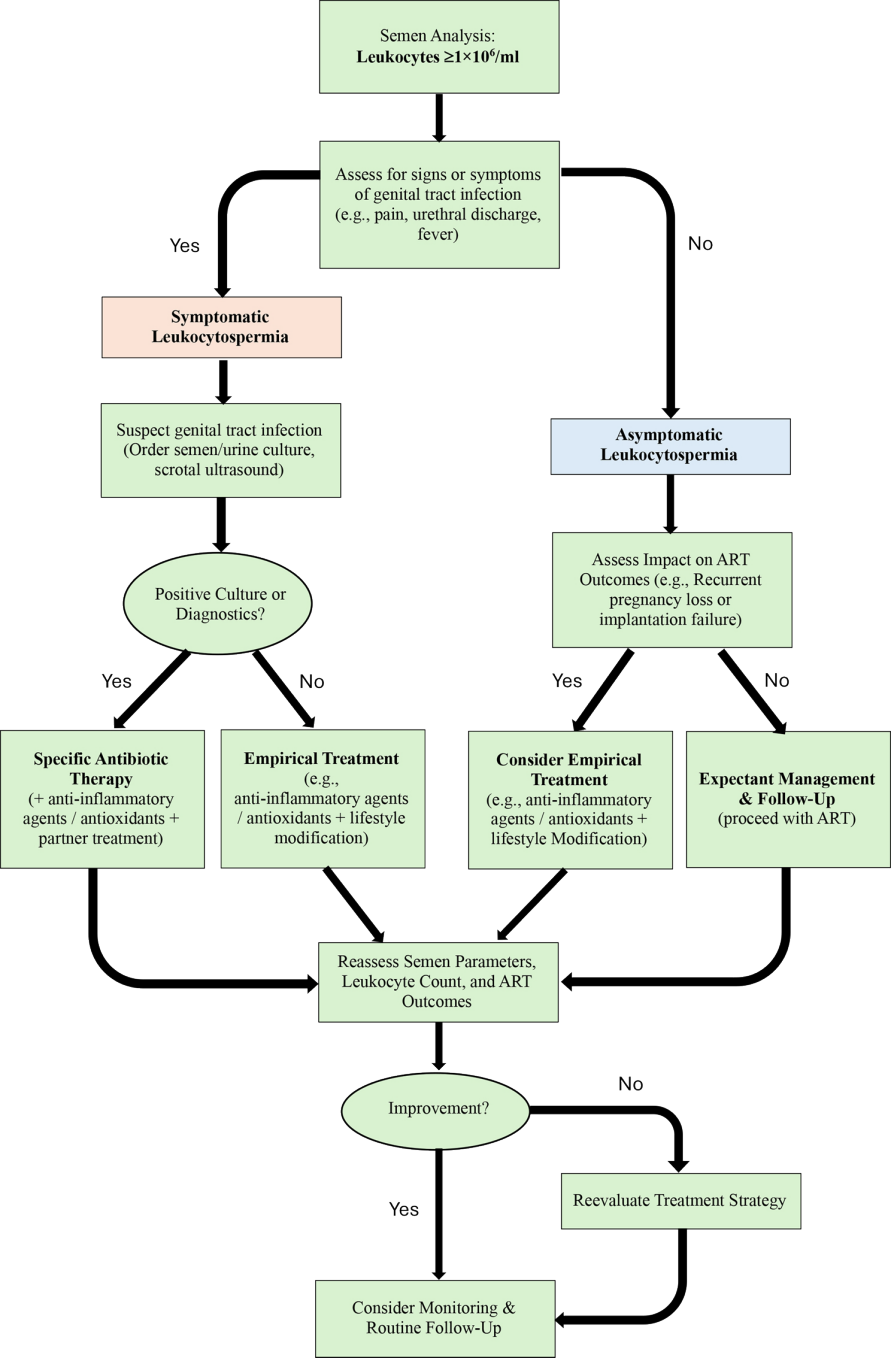
Clinical Management Algorithm for Leukocytospermia in Couples Undergoing Assisted Reproductive Technology.

While routine treatment is not recommended, selective intervention may be justified in specific cases (Figure-2, Table-1), as listed below.

**Table 1:** Management of Leukocytospermia in Male Partners of Couples Undergoing Assisted Reproductive Technology.

Parameter	Symptomatic Leukocytospermia	Asymptomatic Leukocytospermia
**Diagnostic Approach**	Detailed medical history and physical examination Comprehensive evaluation of symptoms (e.g., pain, fever, dysuria, swelling), urine analysis, and imaging studies as appropriate Microbiological analysis to determine the causative pathogen (e.g., semen culture, Gram staining, PCR) (38, 97)	Detailed medical history and physical examination Screening* based on semen analysis during fertility evaluation
**Primary Treatment**	Antibiotic therapy based on identified pathogen and infection site (37, 38, 97)	Expectant management Consider lifestyle modifications (e.g., smoking cessation, weight loss) (97)
**Anti- inflammatory Treatment**	NSAIDs or COX-2 inhibitors may be considered (99) alongside antibiotic therapy, particularly if symptoms	NSAIDs or COX-2 inhibitors may be considered to improve sperm parameters (5, 15)
**Antioxidants**	May be considered alongside antibiotic therapy (14, 103), particularly if markers of oxidative stress present (e.g., high sperm DNA fragmentation levels)	May be considered to improve sperm parameters (97), particularly if markers of oxidative stress present (e.g., high sperm DNA fragmentation levels)
**Surgical Treatment**	Treatment of obstructive causes should be considered (e.g., partial ejaculatory duct obstruction) (13, 97)	May may be considered if clinical varicocele is present (13, 97)
**Duration of Treatment**	Depends on etiology; typically, 1-4 weeks for antibiotic therapy (8)	Not determined
**Follow-up**	Repeat semen analysis* and microbiological analysis post- treatment to confirm leukocytospermia and infection resolution	Repeat semen analysis to confirm leukocytospermia resolution* Consider microbiological analysis to assess subclinical infection if ART failure or miscarriage

ART = assisted reproductive technology; PCR = polymerase chain reaction; ROS = reactive oxygen species; IVF = in vitro fertilization; ICSI = intracytoplasmic sperm injection; NSAIDs = non-steroidal anti-inflammatory drugs; COX = cyclooxygenase; reference numbers are shown in parentheses.

* Screening tools such as the peroxidase test or immunocytochemical staining with CD45 may be used.

1. Symptomatic Male Genital Tract Infections (MGTI)
  - Antibiotic therapy is recommended if bacterial infection is confirmed as the likely cause of leukocytospermia (37, 38, 97).
  - In cases of sexually transmitted infections, partner treatment should also be considered (38, 101, 102).
2. Adjunctive Therapies
  - Antioxidants (e.g., Vitamin C, E, Selenium, Coenzyme Q10) may help reduce oxidative stress without the risks of antibiotics (14, 103, 104), though their impact on fertility outcomes remains uncertain (37, 38).
  - Anti-inflammatory therapy (e.g., COX 2 inhibitor) may improve semen parameters and reduce leukocytospermia, but the effect on pregnancy rates remains inconclusive (5, 15).
3. Recurrent ART Failure
  - We suggest that in cases of recurrent early pregnancy loss and implantation failure, it may be justifiable to alleviate leukocytospermia using antibiotics, anti-inflammatory drugs, or antioxidants.

### Future Directions

Despite advancements in understanding leukocytospermia, several knowledge gaps remain, particularly in distinguishing symptomatic from asymptomatic cases and assessing the possible impact on reproduction by comparing asymptomatic leukocytospermic infertile men with fertile controls. Future research should prioritize the standardization of leukocyte detection methods, such as flow cytometry versus peroxidase staining, to enhance diagnostic accuracy and clinical relevance. Additionally, further studies are needed to evaluate the efficacy of targeted treatment approaches, if any, including antioxidants and anti-inflammatory therapies, in improving ART outcomes for leukocytospermic patients.

## CONCLUSIONS

Current evidence suggests that among couples undergoing ART, asymptomatic leukocytospermia does not compromise the outcomes and should, therefore, not be routinely treated, mainly when semen processing methods are used (e.g., density gradient centrifugation), and ICSI is the fertilization method. While some studies indicate a potential association between leukocytospermia and an increased risk of early pregnancy loss and ectopic pregnancy, these findings are based on low-quality evidence and require further investigation. For now, we suggest that clinical decisions regarding the treatment of asymptomatic leukocytospermia should be individualized, particularly in cases of recurrent early pregnancy loss and unexplained ART failure. Until more robust data emerge, a pragmatic, evidence-based approach remains crucial in balancing the potential risks and benefits of treatments to alleviate leukocytospermia in the context of ART.
